# Long noncoding RNA NEAT1 promotes cardiac fibrosis in heart failure through increased recruitment of EZH2 to the Smad7 promoter region

**DOI:** 10.1186/s12967-021-03211-8

**Published:** 2022-01-03

**Authors:** Zhuowang Ge, Chengye Yin, Yingze Li, Ding Tian, Yin Xiang, Qianhui Li, Yong Tang, Yachen Zhang

**Affiliations:** 1 Department of Cardiology, Xinhua Hospital, School of Medicine, Shanghai Jiao Tong University, 1665 Kongjiang Road, Shanghai, 200092 China; 2 Department of Geriatrics, Zhongda Hospital, School of Medicine, Southeast University, Nanjing, 210009 China

## Abstract

Cardiac fibrosis, a well-known major pathological process that ultimately leads to heart failure, has attracted increasing attention and focus in recent years. A large amount of research indicates that long noncoding RNAs (lncRNAs) play an important role in cardiac fibrosis, but little is known about the specific function and mechanism of the lncRNA NEAT1 in the progression of cardiac fibrosis to heart failure. In the present study, we have demonstrated that the lncRNA NEAT1 is upregulated in patients with heart failure. Similarly, the expression of Neat1 was also increased in the left ventricular tissue of transverse aortic constriction (TAC) surgery mice and cardiac fibroblasts treated with TGF-β1. Further, gain-of-function and loss-of-function experiments showed that silencing of Neat1 attenuated cardiac fibrosis, while overexpression of Neat1 with adenovirus significantly aggravated the in vitro progression of fibrosis. With regard to the underlying mechanism, our experiments showed that Neat1 recruited EZH2 to the promoter region of Smad7 through physical binding of EZH2 to the promoter region, as a result of which Smad7 expression was inhibited and the progression of cardiac fibrosis was ultimately exacerbated. We found that the introduction of shNeat1 carried by adeno-associated virus-9 significantly ameliorated cardiac fibrosis and dysfunction caused by TAC surgery in mice. Overall, our study findings demonstrate that the lncRNA Neat1 accelerates the progression of cardiac fibrosis and dysfunction by recruiting EZH2 to suppress Smad7 expression. Thus, NEAT1 may serve as a target for the treatment of cardiac fibrosis.

**Supplementary Information:**

The online version contains supplementary material available at 10.1186/s12967-021-03211-8.

## Introduction

Despite the major developments in medical science, the mortality and morbidity rates associated with heart failure remain high [1–3]. Further, despite the growing body of research on the causes and treatment of heart failure, the specific pathophysiological mechanisms are still unclear. Among the known mechanisms, cardiac fibrosis, characterized by abnormal excessive deposition of extracellular matrix, is considered to be a key process in the link between ventricular remodeling and heart failure [4–6]. Therefore, it is widely agreed that targeting cardiac fibrosis is a promising strategy for the treatment of heart failure [7–9].

Long noncoding RNAs (lncRNAs) are a subtype of noncoding RNAs that are longer than 200 nucleotides [10, 11]. lncRNAs have gained attention for their role in various biological processes, including cell migration, proliferation, and autophagy [12–14]. Recently, an increasing number of studies have demonstrated the involvement of several lncRNAs in fibrosis-related diseases in a variety of organs, including the heart, lung, liver, kidney, and skin [15–19]. Accordingly, it is now generally accepted that lncRNAs have immense potential in the treatment of fibrosis-related organ diseases.

The lncRNA nuclear enriched abundant transcript 1 (NEAT1) plays an essential role in paraspeckles [20, 21]. NEAT1 has also been reported to be involved in various physiological processes, including tumorigenesis, myogenesis, sepsis, liver failure, and non-alcoholic steatohepatitis [22–26]. In addition, some researchers have reported that NEAT1 is associated with fibrosis-related diseases. For example, Zhang et al. demonstrated that NEAT1 modulates pulmonary fibrosis via the TGF-β pathway [27]. Yu et al. reported that NEAT1 could accelerate the progression of liver fibrosis [28], and Li et al. showed that NEAT1 sponges miR-129 to regulate renal fibrosis [29]. However, so far, the function and specific mechanisms of NEAT1 in cardiac fibrosis associated with heart failure remain largely unknown.

In the present study, we have explored the role of Neat1 in regulating cardiac fibrosis through a set of in vitro and in vivo experiments. Our findings indicate that NEAT1/Neat1 was highly upregulated in the cardiac tissue of heart failure patients and mouse models of heart failure. Further, both overexpression and knockdown experiments showed that Neat1 could exacerbate cardiac fibrosis. With regard to the underlying mechanism, our findings showed that Neat1 promoted the progression of cardiac fibrosis to heart failure by recruiting EZH2 to the promoter region of Smad7. In summary, our results indicate that NEAT1 may be a novel target for the treatment of cardiac fibrosis and heart failure.

## Materials and methods

### Ethics statements

C57/BL6J mice were obtained from Xinhua Hospital, Shanghai Jiao Tong University. All the experiments were approved by the ethics committee of Shanghai Jiao Tong University. The procedures were performed according to the Guide for the Care and Use of Laboratory Animals (NIH Publications, 1996).

### Human left ventricle samples

Left ventricle samples of the resected heart were obtained from patients (n = 6) with dilated cardiomyopathy (DCM) who had undergone heart transplantation. Normal heart specimens were obtained from donors (n = 6) who died in accidents whose hearts were unsuitable for transplantation for non-cardiac reasons. The collection and use of all human left ventricle samples were approved by the local human research ethics committee of Shanghai Xinhua Hospital, Shanghai Jiao Tong University School of Medicine, and were in accordance with the Declaration of Helsinki.

### Establishment of model mice

Transverse aortic constriction (TAC) model mice were established surgically using a previously reported method [30]. The surgeries were carried out on 8-week-old male mice. In brief, a 6–0 suture was placed in the ascending aorta and tightened around a 27-gauge needle. When the needle was removed, the aorta contracted to a uniform diameter. During the sham operation, the same procedure was performed without tying of the sutures.

### Isolation and culture of cardiac fibroblasts

Primary culture of cardiac fibroblasts (CFs) was carried out as described previously [31]. Briefly, CFs were isolated from C57/BL6J mice born in three days. Next, hearts were cut into small pieces and digested using 0.05% (w/w) trypsin/EDTA (Gibco) and 0.05% (w/w) type II collagenase (Invitrogen) at 37 °C. The digested sample was then centrifuged and resuspended, and the cells were plated on complete Dulbecco’s modified Eagle’s medium (Gibco BRL, USA) containing 10% fetal bovine serum (Gibco BRL), penicillin, and streptomycin (100 mg/mL). The medium was changed every 2 days. The primary CFs were incubated until the cells reached 70–80% fusion on the plate before they were used in the experiments.

### siRNA transfection

Neat1 siRNA and negative control siRNA were purchased from Ribobio (Guangzhou, China). The siRNAs were transfected into two different groups of cells that had reached 70% confluence via Opti-MEM medium and Lipofectamine 2000 (Invitrogen). After 24 h of transfection, the cells were collected to quantify the expression of target genes.

### In vitro transfection of adenovirus vectors and in vivo transfection of adeno-associated virus 9 vectors

Two recombinant adenoviral vectors based on the pDC315-EGFP vector (purchased from Hanbio Co. Ltd., Shanghai, China) were constructed to express the mouse Neat1 gene (Ad-Neat1) and the green fluorescence protein gene (Ad-GFP, control), under the control of the cytomegalovirus promoter. The stock solutions of Ad-Neat1 and Ad-GFP were at concentrations of 1.25 × 10^11^ and 2.50 × 10^11^ plaque formation units (PFUs)/ml, respectively. A working solution of 1 μl of vehicle containing 1.0 × 10^9^ PFU. The transfection efficiency of Neat1-overexpression adenovirus was determined using GFP analysis (Additional file 1: Fig. S1A).

Neat1-shRNA virus (pAAV-U6-shRNA(Neat1)-WPRE) and controls were constructed by Obio Technology. Neat1-shRNA was also constructed by Obio Technology (the target sequence of Neat1-shRNA is GCGCAAGTTAGCCACAAAT). We generated adeno-associated virus-9 (AAV-9) carrying shNeat1 (to silence Neat1 expression) and control scramble plasmids, and introduced them into 4-week-old mice via tail vein injection. At 4 weeks after the injection, the mice were subjected to TAC surgery or sham surgery and sacrificed at 8 weeks after the surgery.

### Echocardiographic measurements of the left ventricle

At 8 weeks after TAC surgery, echocardiography was performed with an ultrasound instrument (Vivid7, GE Healthcare). Mice were then anesthetized with 2% isoflurane inhalation and placed on a heated pad to maintain a body temperature of 37℃. The left ventricle end-diastolic diameter, left ventricle end-systolic diameter, and left ventricle ejection fraction were measured.

### Transwell invasion assay

A 24-well transwell chamber (Corning Incorporation, NY, USA) was used to examine cell invasion. CFs were seeded into the upper chamber at a density of 4 × 10^4^ cells. After incubation at 37 °C for 24 h, the cells that had migrated to the lower chamber were observed and counted under a microscope (Leica). Finally, five fields of 200 × magnification were used to count the number of migrating fibroblasts per field.

### RT-qPCR

The expression of Neat1, PCNA, CyclinD1, and P27Kip1 mRNA was detected with RT-qPCR as previously reported. Briefly, total RNA from samples were extracted with the TRIzol kit (Invitrogen, USA), and the concertation of RNA was measured using Nanodrop2000 (Thermo Fisher, MA). cDNA was synthesized using the ReverAid First Strand cDNA Synthesis kit (Thermo, USA) and Primescript RT Master Mix kit (Takara, Japan). RT-qPCR was conducted with the SYBR Green PCR kit (TAKARA, Japan) in a Real-Time PCR Detection System (BioRad, USA). The 2-ΔΔCt method was applied to calculate relative mRNA expression.

### Western blotting analysis

Protein expression in cultured cells was detected with western blotting as previously reported. Briefly, cultured myocardial cells or tissue homogenate was harvested, and total proteins were extracted with the RIPA buffer (Beyotime, China). The BCA protein assay kit (Thermo Scientific) was then used to detect the protein concentration in the samples. SDS-PAGE was applied to separate the proteins, and the separated proteins were transferred to a PVDF membrane that was then blocked with 5% non-fat milk. Next, the proteins on the PVDF membranes were labeled with primary antibodies against α-SMA (Abcam, ab32575), Collagen1 (Abclonal, A1352), Collagen3 (Abcam, ab7778), CTGF (Abcam, ab6992), EZH2 (CST, #5246S), H3K27me3 (Abclonal, A2363), p-Smad2/3 (Abclonal, AP0548), T-Smad2/3 (Abclonal, A7536), Smad7 (Abclonal, A16396), and GAPDH (Abcam, ab181602), and then the corresponding secondary antibodies (Sigma). The ECL kit was used to visualize the blots. Images of the blots were obtained and analyzed using Image-Pro-Plus 6.0. The relative expression of proteins was determined by normalizing their expression to the expression of GAPDH.

### Immunohistochemical analysis

Cardiac tissue samples were fixed with 10% formalin phosphate buffer for 24 h, embedded in paraffin, and cut into 4-µm sections for immunohistochemical and immunofluorescence staining. Picrosirius red (PSR) staining was performed according to the manufacturer’s instructions to determine the degree of cardiac fibrosis and the percentage of collagen-positive (red) area in the total tissue area. Immunofluorescence staining was conducted using antibodies against vimentin (Abcam, ab92547). Cross-sectional areas of individual cells were visualized using an Olympus DP80 microscope. All the images were acquired and analyzed by Image-Pro Plus 6.0 as previously described [32].

### RNA-binding protein immunoprecipitation assay

The Magna RIP™ Quad RNA-Binding Protein Immunoprecipitation Kit (Millipore) was used for the RNA-binding protein immunoprecipitation (RIP) experiments according to the manufacturer’s instructions. In brief, cultured samples were collected, lysed in RIP lysis buffer, and incubated with EZH2 antibody (CST, #5246S)-coated beads. RNA bound with EZH2 was purified. Neat1 was the predicted binding target, and its level was detected by RT-qPCR (as described earlier).

### Chromatin immunoprecipitation assay

The EZ-Magna ChIP A/G Chromatin Immunoprecipitation kit was used to conduct the chromatin immunoprecipitation (ChIP) assay. As described in the guide, cell lysate was incubated with beads coated with antibodies against EZH2 (CST, #5246S) and H3K27me3 (Abclonal, A2363). The bound DNA fragments were eluted and measured by RT-qPCR to determine the enrichment of EZH2 or H3K27me3 in the promoter region of the target genes.

### Statistical analysis

The data are expressed as mean ± standard error. All statistical analyses were performed using GraphPad Prism 6.0. One-way analysis of variance (ANOVA) followed by the Tukey test was used to analyze differences among groups. All the experiments were conducted in duplicate. P < 0.05 was considered to indicate statistical significance.

## Results

### Upregulation of NEAT1 expression in left ventricle tissue samples of DCM patients

**Fig. 1:**
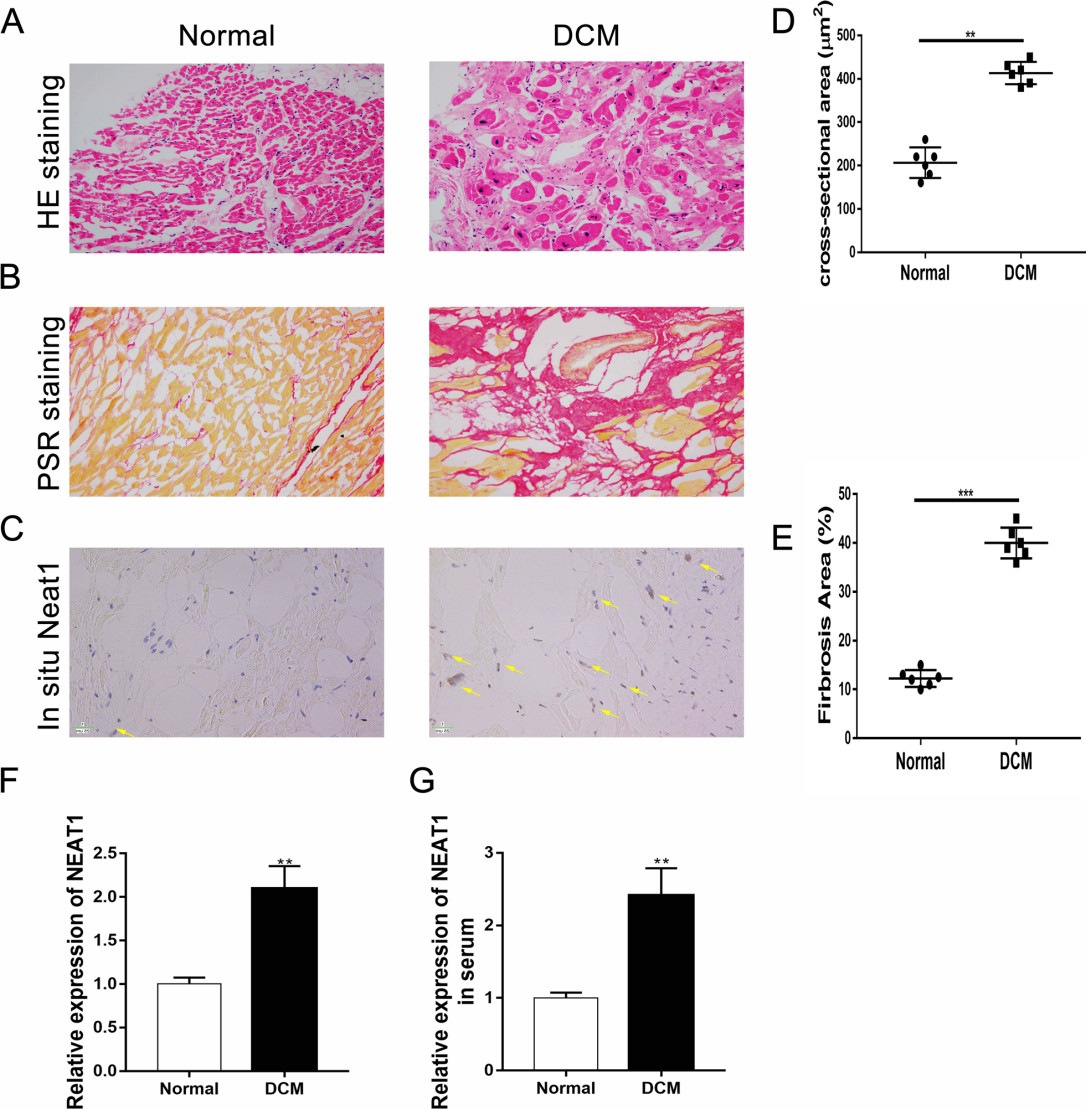
Severe fibrosis and increased expression of NEAT1 in the left ventricular tissue of DCM patients. **A**–**C** Representative HE staining, PSR staining, and in situ hybridization results for Neat1 (scale bar = 20 µm; n = 6 in each group). **F**, **G** Quantitative polymerase chain reaction results for mRNA expression of Neat1 in the left ventricle (n = 6 in each group) or serum (n = 20 in each group) obtained from patients with DCM and control patients. Data are presented as mean ± SEM. **p < 0.01 vs. the normal group, ***p < 0.001 vs. the normal group

HE staining and PSR staining of myocardial tissue samples from DCM patients showed obvious morphological and structural changes that were indicative of significant fibrosis (Fig. 1A, B). These changes are quantified in Fig. 1D, E). In addition, we examined the expression level and localization of NEAT1 by immunostaining and fluorescence in situ hybridization analysis. The results showed that NEAT1 was significantly upregulated in the myocardium of DCM patients (Fig. 1C). Furthermore, the expression of Neat1 in myocardial tissues (Fig. 1F) and serum (Fig. 1G) was higher in the DCM patients than in the normal group. These findings indicate that the level of the lncRNA NEAT1 is elevated in DCM; thus, NEAT1 may play a role in the pathogenesis of DCM.

### Upregulation of Neat1 expression in TAC model mice

**Fig. 2:**
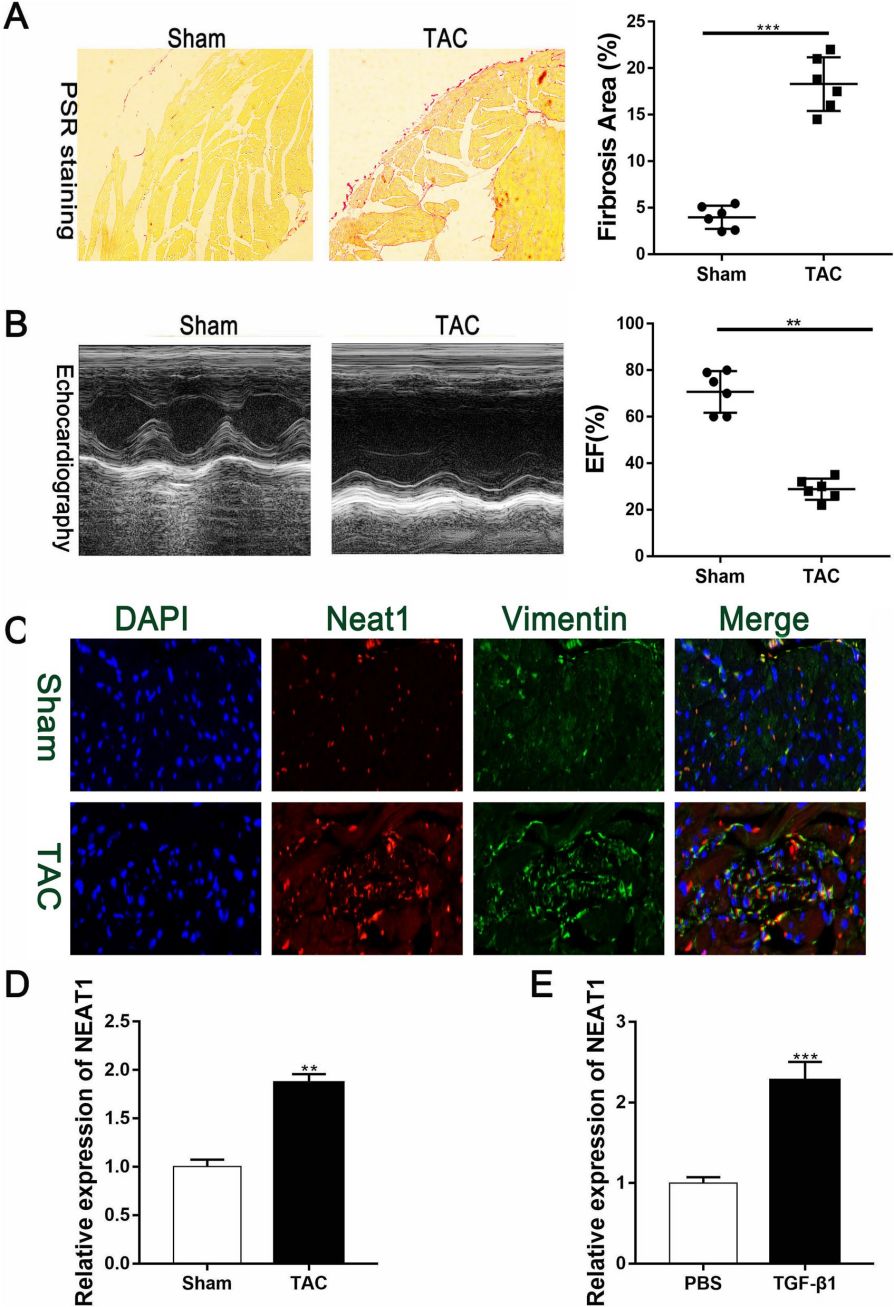
Upregulation of Neat1 in the left ventricular tissue of mice after TAC surgery. **A** Representative PSR staining results were presented and quantified to demonstrate the extent of cardiac fibrosis (n = 6 in each group). **B** Representative M-mode images of TAC and sham group mice and quantification of EF% via echocardiography (n = 6 in each group). **C** Representative immunofluorescence images and quantitative data for Neat1 expression in the left ventricles (n = 8 in the sham group and n = 10 in the TAC group; red, Neat1; green, vimentin; blue, DAPI; scale bar = 50 µm). **D** Expression of Neat1 according to qRT-PCR results in the sham group or TAC group (n = 6 in each group). **E** Expression of Neat1 in PBS-treated CFs or TGF-β1-treated CFs (n = 6 in each group). Data are presented as mean ± SEM. **p < 0.01, ***p < 0.001 for differences between the indicated groups

Numerous studies have reported that mice present with significant cardiac fibrosis after TAC surgery. Accordingly, compared with the sham group, obvious cardiac fibrosis was observed in the cardiac tissue of the TAC mice after PSR staining (Fig. 2A). Echocardiography showed that the left ventricular ejection fraction was significantly reduced in mice after the TAC surgery (Fig. 2B). We also analyzed the expression of Neat1 and vimentin, a cardiac fibrosis marker, by immunofluorescence staining and found that Neat1 and vimentin were both upregulated in the areas with cardiac fibrosis (Fig. 2C). RT-PCR results also showed that the expression of Neat1 was markedly upregulated in cardiac tissue after TAC surgery (Fig. 2D). Further, primary CFs pretreated with TGF-β1 for 48 h also showed high Neat1 expression (Fig. 2E). Thus, both the in vivo and in vitro findings demonstrate that expression of the lncRNA Neat1 is upregulated during the progression of cardiac fibrosis.

### In vitro inhibition of TGF-β1-induced cardiac fibrosis by Neat1 knockdown

**Fig. 3:**
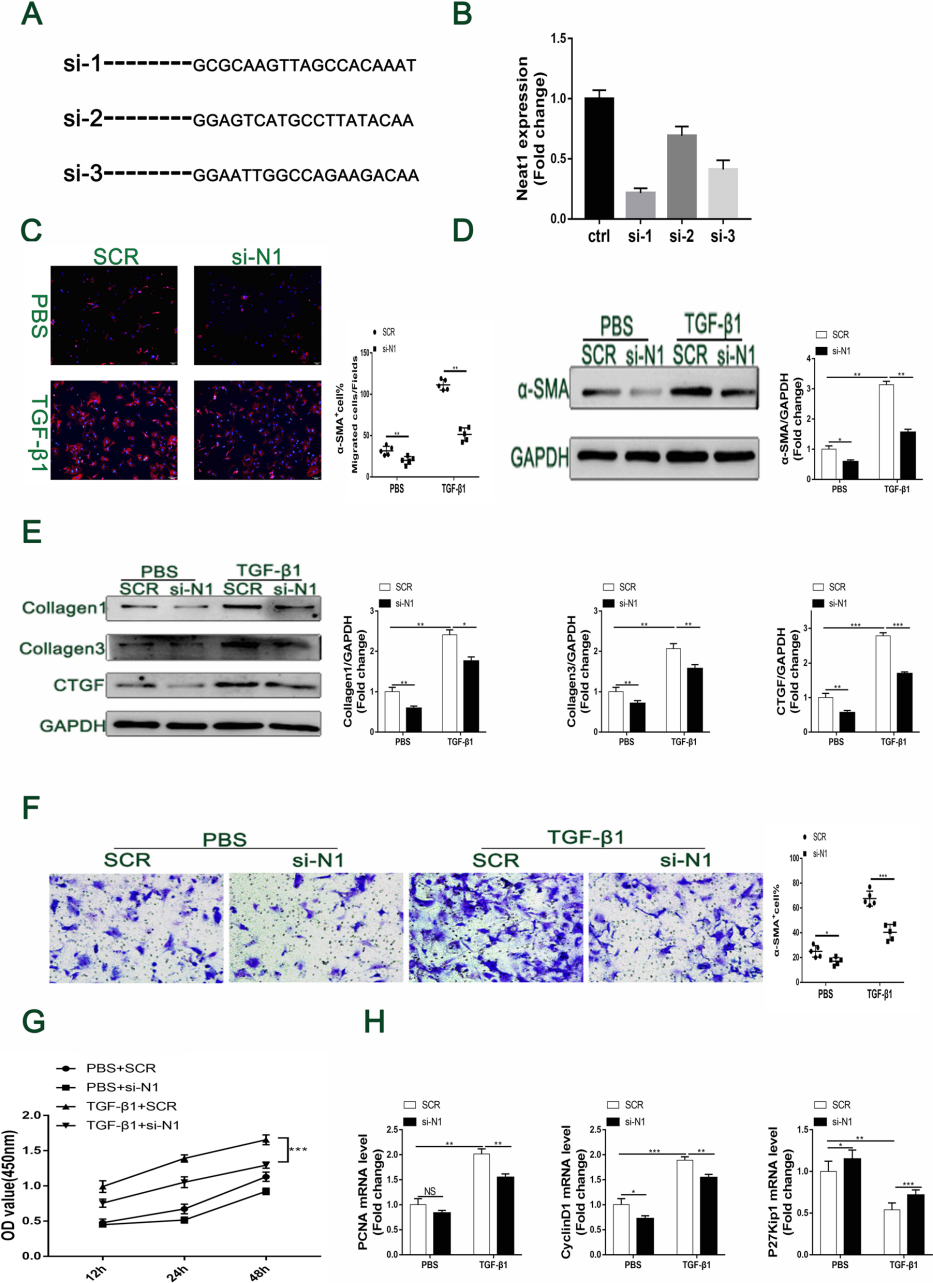
Alleviation of TGF-β1 induced cardiac fibrosis, migration, and proliferation in cultured CFs by siRNA-induced Neat1 silencing. **A** Specific sequences of the three constructed siRNAs against Neat1. **B** Knockdown efficiency of the three different siRNAs against Neat1 was verified by RT-PCR (n = 4 in each group). **C** CFs were analyzed by immunofluorescence analysis of the expression of α-SMA (red) and nuclei (DAPI: blue) (n = 5 in each group; scale bar = 500 µm). **D** Protein level and quantification of α-SMA were determined by western blot analysis after TGF-β1 treatment for 48 h (n = 5 in each group). **E** Relative protein levels and quantification of extracellular matrix synthesis-associated proteins in cultured CFs of the indicated groups (n = 5 in each group). **F** Representative images of Transwell migration assay and quantification of migrated CFs in the indicated groups (n = 5 in each group; scale bar = 100 µm). **G** Quantification by the CCK-8 assay (n = 5 in each group). **H** mRNA levels of PCNA, cyclin D1, and P27 Kip1 by qRT-PCR (n = 5 in each group). Data are presented as mean ± SEM. *p < 0.05, **p < 0.01, ***p < 0.001, NS = no significant difference between the indicated groups

To determine the biological function of Neat1 in TGF-β1-induced cardiac fibrosis, we synthesized siRNAs against Neat1 and transfected primary CFs with the siRNAs (Fig. 3A). The qPCR results illustrated that the first sequence of synthetic siRNAs against Neat1 could most effectively decrease the expression of Neat1 in cardiac fibroblasts (Fig. 3B). Thus, the following experiments were carried out with si-1-Neat1. Immunofluorescence staining and western blotting results both showed that silencing Neat1 obviously decreased the expression of α-SMA, which is a myofibroblast marker of the phenotypic switch from fibroblasts to myofibroblasts (Fig. 3C, D). Furthermore, the protein levels of TGF-β1-induced fibrotic markers, including collagen 1/3 and CTGF, were all downregulated in the si-1-Neat1 group (Fig. 3E). Additionally, silencing Neat1 dramatically alleviated TGF-β1-induced cell proliferation and migration, as indicated by the results of the Transwell migration assay and CCK8 assay (Fig. 3F, G). Analysis of proliferation markers (Fig. 3H) showed that upregulation of proliferating cell nuclear antigen (PCNA) and cyclin D1 by TGF-β1 stimulation was significantly alleviated by si-Neat1. On the contrary, the expression of p27Kip1 (an inhibitor of a cyclin-dependent kinase) was increased in Neat1-siRNA-treated cardiac fibroblasts. These results indicate that the knockdown of Neat1 attenuated TGF-β1-induced cardiac fibrosis.

### In vitro promotion of TGF-β1-induced cardiac fibrosis by Neat1 overexpression

**Fig. 4:**
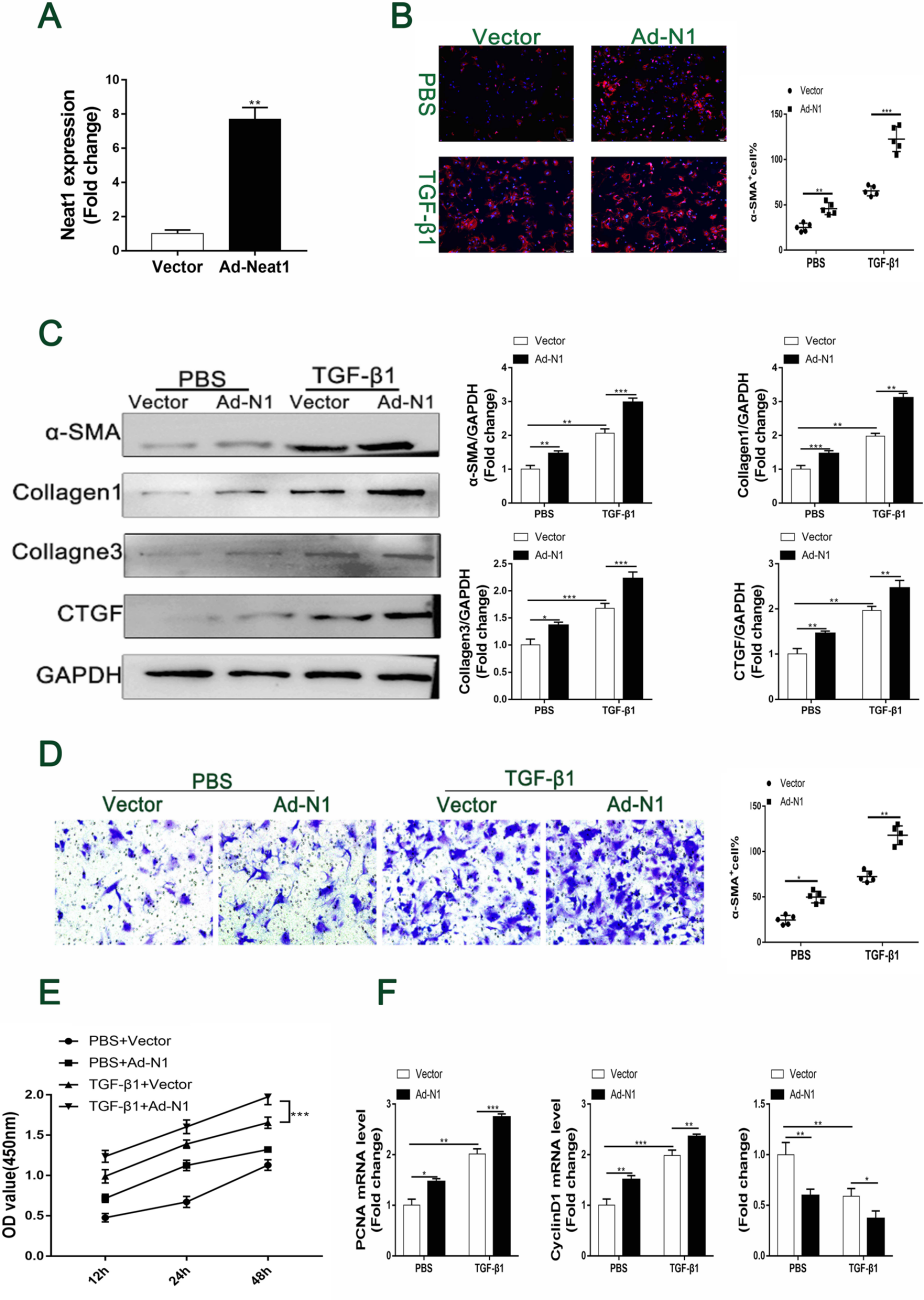
Promotion of cardiac fibrosis, migration, and proliferation in cultured CFs by adenovirus-induced Neat1 overexpression. **A** The overexpression efficiency of Ad-Neat1 was demonstrated by q-PCR (n = 4 in each group). **B** CFs were analyzed by immunofluorescence for the expression of α-SMA (red) and nuclei (DAPI: blue) (n = 5 in each group; scale bar = 500 µm). **C** Representative western blotting and quantitative analysis of α-SMA and ECM-related proteins in CFs in the indicated groups (n = 5 in each group). **D** Representative images of Transwell migration assay and quantification of migrated CFs for the indicated groups (n = 5 in each group; scale bar = 100 µm). **E** Quantification of proliferation by the CCK-8 assay (n = 5 in each group). **F** mRNA levels of PCNA, cyclin D1, and P27 Kip1 by qRT-PCR (n = 5 in each group). Data are presented as mean ± SEM. *p < 0.05, **p < 0.01, ***p < 0.001, NS = no significant difference between the indicated groups

To further investigate the role of the lncRNA Neat1 in TGF-β1-induced cardiac fibrosis, we induced Neat1 overexpression in TGF-β1-treated CFs with a Neat1-expressing adenovirus vector (Fig. 4A). Immunofluorescence staining showed that Neat1 overexpression led to a considerable increase in the expression of α-SMA; this means that it enhanced TGF-β1-induced phenotypic switching (Fig. 4B). Similarly, the protein levels of α-SMA, collagen 1/3, and CTGF were all upregulated in the Neat1-overexpression group (Fig. 4C). Furthermore, Neat1 overexpression significantly enhanced TGF-β1-induced cell proliferation and migration (Fig. 4D, E). Similarly, expression of the proliferation markers PCNA and cyclin D1 was dramatically increased by Neat1 overexpression. In contrast, the expression level of p27Kip1 was prominently decreased (Fig. 4F). Collectively, the results of these in vitro experiments show that Neat1 promotes TGF-β1-induced cardiac fibrosis.

### Promotion of cardiac fibrosis by Neat1 via recruitment of EZH2

**Fig. 5:**
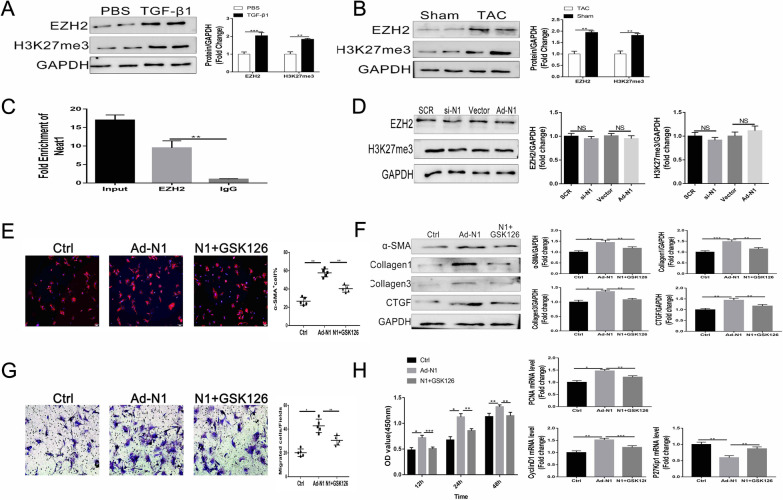
Aggravation of cardiac fibrosis by Neat1 via recruitment of EZH2. **A**, **B** Representative western blotting and quantitative analysis findings for EZH2 and H3K27me3 in CFs (n = 5 in each group). **C** Interaction between Neat1 and EZH2 verified by the RIP assay (n = 5 in each group). **D** Representative western blotting and quantitative analysis findings for EZH2 and H3K27me3 in CFs (n = 5 in each group). **E** CFs were analyzed by immunofluorescence for the expression of α-SMA (red) and nuclei (DAPI: blue) (n = 5 in each group; scale bar = 500 µm). **F** Representative western blotting and quantitative analysis of α-SMA and ECM-related proteins in CFs (n = 5 in each group). **G** Representative images of Transwell migration assay and quantification of migrated CFs (n = 5 in each group; scale bar = 100 µm). **H** Quantification of cell proliferation by the CCK-8 assay (n = 5 in each group) and mRNA levels of PCNA, cyclin D1, and P27 Kip1 by qRT-PCR. Data are presented as mean ± SEM. *p < 0.05, **p < 0.01, ***p < 0.001, NS = no significant difference between the indicated groups

We first learned from the previous studies that some LncRNAs can bind to EZH2 to regulate diverse diseases [25, 26, 33], and that EZH2 happened to be reported to be involved in some cardiovascular-related diseases progression in recent years[32, 34]. So, we started to hypothesize whether Neat1 also regulated the progression of cardiac fibrosis through EZH2. And before proceeding to a specific experimental validation, we used two bioinformatics analysis websites beforehand to demonstrate the potential binding possibility between NEAT1 and EZH2, and the results of both websites suggested a high possibility of Neat1 binding to EZH2 (Preliminary prediction by bioinformatics system showed that NEAT1 may have certain binding ability to EZH2 (http://service.tartaglialab.com/page/catrapid_group) (Additional file 3: Fig S3A). In addition, we predicted the interaction probabilities of NEAT1and EZH2 via RNA–Protein Interaction Prediction (RPISeq) (http://pridb.gdcb.iastate.edu/RPISeq/results.php) and found that Neat1 could potentially bind to EZH2 (Additional file 3: Fig S3B). Interestingly, we found the protein levels of EZH2 and H3K27me3 were significantly upregulated in TGF-β1-treated CFs (Fig. 5A), as well as in the cardiac tissue samples of TAC mice (Fig. 5B). Importantly, the RIP assay and pull-down experiment (Additional file 3: Fig S3) demonstrated the binding of EZH2 with Neat1 (Fig. 5C). However, the levels of EZH2 and its methylation site H3K27me3 were unaffected by the expression of Neat1 (Fig. 5D). The immunofluorescence staining results indicated that GSK126 (an EZH2 inhibitor) could reverse the increased expression of α-SMA in CFs induced by Neat1 overexpression (Fig. 5E). That is, the increased protein levels of α-SMA, collagen 1/3, and CTGF in Neat1-treated CFs were partly offset by GSK126. This trend was also observed with regard to cell proliferation and migration (Fig. 5G, H). All these results imply that Neat1 promotes cardiac fibrosis by recruiting EZH2, without affecting the EZH2 expression level.

### Role of the Smad7 signaling pathway in the EZH2-mediated pro-fibrotic function of Neat1

**Fig. 6:**
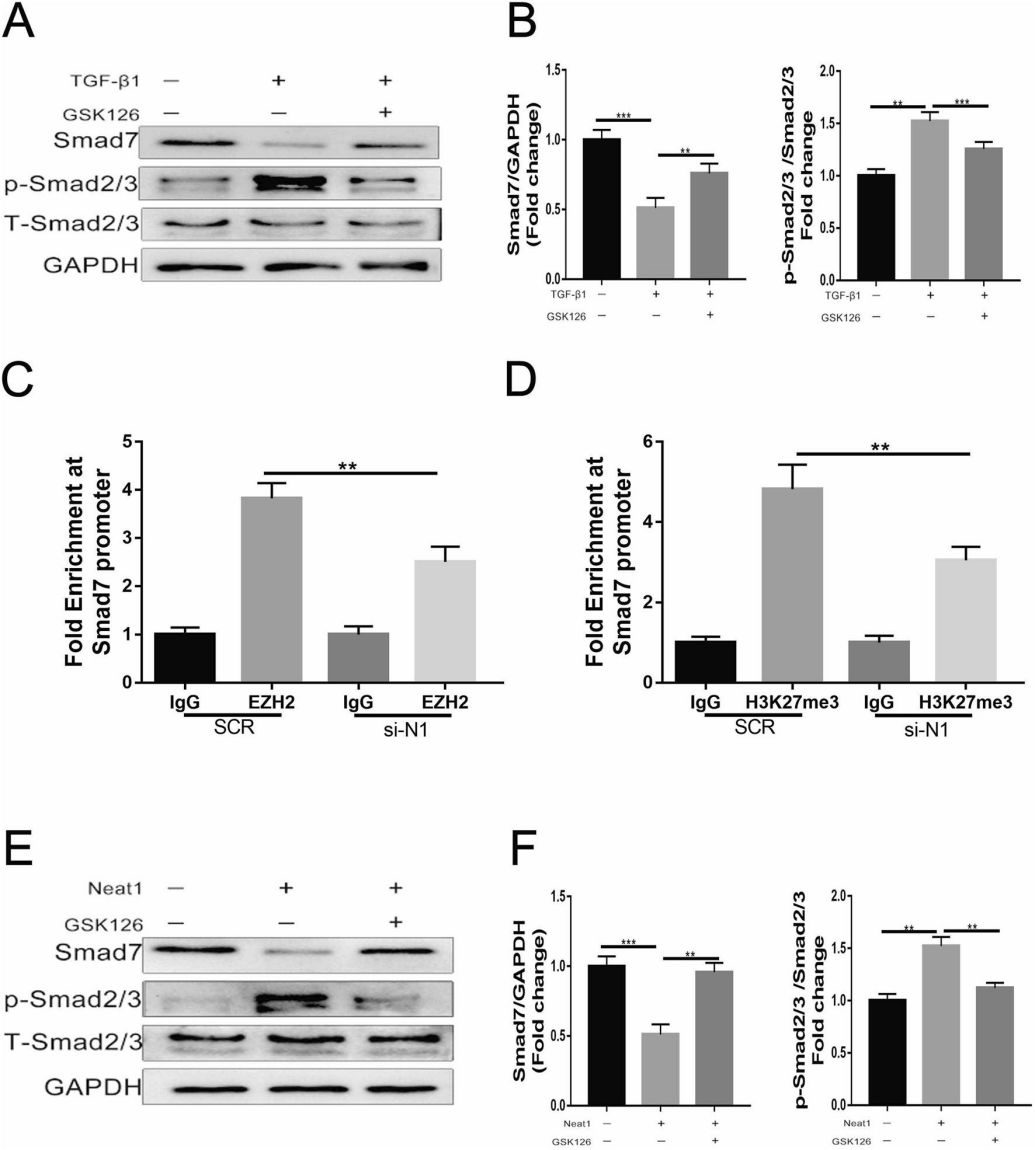
Neat1-mediated binding of EZH2 to the Smad7 promoter region. **A**, **B** Expression of Smad7 and p-Smad2/3 was assessed by western blotting and quantified relative to GAPDH and total Smad2/3 expression (n = 5 in each group). **C**, **D** Enrichment of EZH2 and H3K27me3 on the Smad7 promoter region determined by ChIP-qPCR (n = 5 in each group). **E**, **F** Western blotting analysis and quantification of the expression of Smad7 and p-Sma2/3 (n = 5 in each group). Data are presented as mean ± SEM. *p < 0.05, **p < 0.01, ***p < 0.001, NS = no significant difference between the indicated groups

To further study the molecular mechanism by which Neat1 regulates cardiac fibrosis, we investigated the expression of Smad7, which has been found to be inhibited by EZH2 binding. The protein level of Smad7 was downregulated in TGF-β1-treated CFs, while Smad2/3 was upregulated. As expected, the EZH2 inhibitor GSK126 remarkably alleviated Smad7 downregulation and Smad2/3 upregulation caused by TGF-β1 (Fig. 6A, B). Furthermore, ChIP-qPCR experiments demonstrated that EZH2 could bind to the promoter of Smad7, and the extent of the binding was partly regulated by Neat1 (Fig. 6C). The ChIP-qPCR results for H3K27me3 were similar (Fig. 6D). Additionally, GSK126 reversed the downregulation of Smad7 expression and the upregulation of Smad2/3 caused by the overexpression of Neat1 (Fig. 6E, F). Besides, we applied Smad7 overexpression adenovirus, while giving NEAT1 overexpression adenovirus at the same time, to validate the fibrosis-related phenotype in an in vitro CFs experiment. We found that overexpression of Smad7 successfully rescued the progression of cardiac fibrosis caused by overexpression of Neat1 (Additional file 1: Figure S1). These findings indicate that Neat1 exerted its pro-fibrotic function by recruiting EZH2 to induce the methylation of histone H3 at the Smad7 promoter to inhibit its expression.

### Effects of Neat1 in TAC model mice

**Fig. 7:**
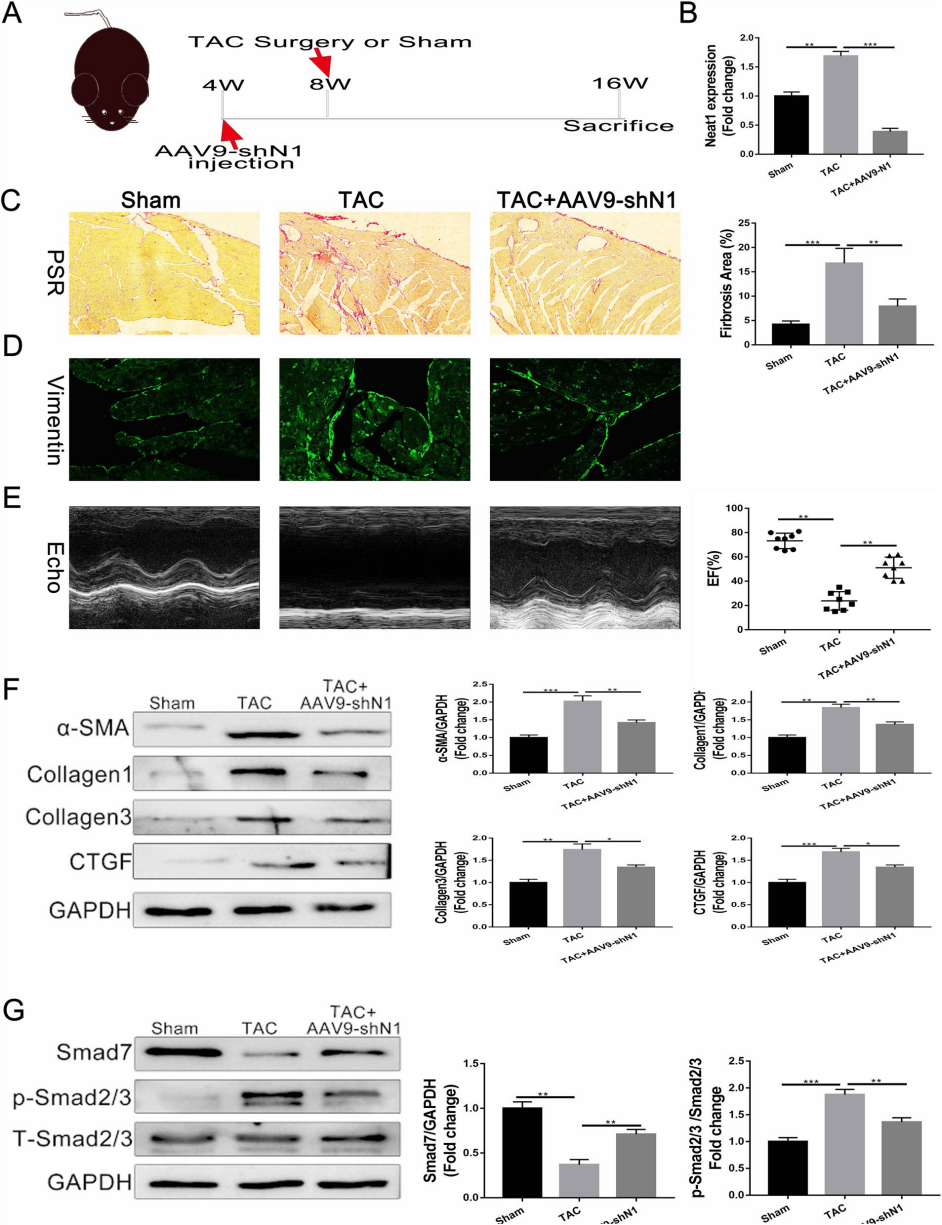
Alleviation of TAC-induced cardiac fibrosis by AAV9-shNeat1. **A** Schema of the procedure for injection of AAV9 carrying shNeat1. **B** The silencing efficiency of AAV9-shNeat1 was verified by q-PCR (n = 6 in each group). **C** Representative results of picrosirius red staining of heart tissues obtained from the indicated groups (n = 7 in each group). **D** Representative immunofluorescence images of collagen I expression in the left ventricular tissue of the indicated groups (n = 7 in each group; scale bar = 100 µm). **E** Representative M-mode images of the indicated groups and EF% quantified by echocardiography (n = 8 in each group). **F**, **G** Representative western blot and quantitative analysis of cardiac fibrosis and Smad7 pathway-related proteins in left ventricular tissues from the indicated groups (n = 6 in each group). Data are presented as mean ± SEM. *p < 0.05, **p < 0.01, ***p < 0.001, NS = no significant difference between the indicated groups

To investigate the in vivo effects of Neat1, shNeat1 carried by AAV-9 was administered in C57/BL6J mice via tail vein injection. At 4 weeks after the injection, mice were subjected to TAC surgery or sham surgery and were sacrificed at week 16 (Fig. 7A). Cardiac tissue was resected, and Neat1 expression was examined. Compared to the control group, in the TAC group, Neat1 expression was upregulated, as described earlier. The administration of shNeat1-AAV significantly attenuated Neat1 upregulation (Fig. 7B) and significantly alleviated the extent of cardiac fibrosis, as demonstrated by PSR staining and immunofluorescence staining for vimentin (Fig. 7C, D). Furthermore, echocardiography results for the left ventricle indicated that the EF% in the shNeat1 group was significantly better than that in the TAC group (Fig. 7E). Accordingly, the levels of the fibrosis markers were reduced in cardiac tissue after shNeat1 administration (Fig. 7F). Similar results were observed for Smad7 signaling pathway-related proteins (Fig. 7G). Taken together, these results indicate that shNeat1 could serve as a potential therapeutic tool for cardiac fibrosis (Additional file 2: Fig. S2).

## Discussion

The lncRNA NEAT1, a non-coding RNA involved in a variety of biological processes [13, 14], was first reported to be associated with a range of tumor-related diseases. It has been found to play a role in tumor proliferation, migration, invasion, and apoptosis in both in vitro and in vivo experiments [35–39]. In recent years, NEAT1 has also been found to be involved in diseases other than tumors, including some fibrosis-related diseases. For example, Li et al. reported that NEAT1 regulated renal fibrosis by sponging miR-129 [29], and Ye et al. and Huang et al. showed that NEAT1 could modulate the progression of liver fibrosis [28, 40]. Additionally, research by Liu et al. and Zhang et al. has demonstrated that NEAT1 promoted pulmonary fibrosis via various pathways [27, 41]. However, there are very few studies on its specific functions in cardiac fibrosis. In this study, we show for the first time that NEAT1/Neat1 expression is significantly elevated in both patients with heart failure and in a mouse model of TAC-induced heart failure. In addition, both gain-of-function and loss-of-function experiments showed that Neat1 plays a role in promoting cardiac fibrosis, and this was confirmed through in vivo experiments on TAC mouse models of heart failure.

There has been increasing interest in the link between Neat1-related pathways and fibrotic diseases, but there are still no detailed reports on the role of Neat1 in cardiac fibrosis. Cardiac fibrosis is a crucial hallmark of heart failure [42] that, ultimately, irreversibly accelerates its clinical progression [43]. Thus, understanding the specific pathogenesis of cardiac fibrosis is essential for designing new therapeutic strategies to prevent heart failure. Interestingly, our findings confirm that inhibition of Neat1 significantly attenuated the adverse progression of cardiac fibrosis and dysfunction, and they are in line with previous reports which showed that Neat1 promoted fibrosis in other organs.

NEAT1 has been reported to be a biomarker for many diseases. Researchers have demonstrated that NEAT1 levels are significantly increased in a variety of tumors, including cholangiocarcinoma [26], colorectal cancer [35], and hepatocellular carcinoma [38]. In addition, Li et al. reported high NEAT1 expression in renal fibrosis [29], and similar results have also been reported for pulmonary fibrosis [27, 41]. Further, a report by Dai et al. revealed that NEAT1 was significantly upregulated in atrial fibrillation. Consistent with the above results, our study confirmed that the expression of NEAT1/Neat1 was significantly increased in heart failure patients and in a TAC-induced fibrosis model in mice. Therefore, NEAT1 might also be a potential novel biomarker for heart failure.

In the present study, we investigated the specific function of Neat1 in cardiac fibrosis. Knockdown of Neat1 expression significantly attenuated the malignant progression of cardiac fibrosis, both under in vivo and in vitro settings, and this was confirmed by the remarkable downregulation of the expression of various fibrosis markers and improved cardiac function. Together, these findings imply that Neat1 plays a role in the malignant progression of cardiac fibrosis. Thus, targeted inhibition of Neat1 might be effective in slowing or halting the progression of cardiac fibrosis to heart failure.

In the present study, we also explored the potential molecular mechanism through which Neat1 exerts its pro-fibrotic effects. The Capture Hybridization Analysis of RNA Targets (CHART) analysis has clarified a group of proteins that might interact with Neat1, thus revealing that Neat1 probably exerts its transcriptional regulatory functions via the mediation of several proteins [44]. We focused on EZH2, which has been reported to be involved in various fibrotic diseases [32, 45–48] and can bind with various lncRNAs, including Neat1, to exert different functions [23, 25, 49]. First, we found that the expression of EZH2 was significantly increased both in TAC mouse tissues and TGF-β1-induced CFs. Although the EZH2 expression pattern was similar to that of Neat1, the expression of EZH2 was not regulated by Neat1. Further, RIP experiments and rescue experiments showed that GSK126, an inhibitor of EZH2, significantly rescued the exacerbation of cardiac fibrosis caused by overexpression of Neat1. These findings demonstrate that Neat1 and EZH2 act via physical binding to promote cardiac fibrosis together.

EZH2 binding to lncRNAs typically occurs at the promoter region of target genes to induce the accumulation of H3K27me3, which in turn epigenetically represses the expression of target genes and exerts a range of effects [23, 25, 49]. Qiang Wang et al. reported that NEAT1 suppressed hepatocyte proliferation by binding to EZH2 and thereby inhibiting the transcriptional level of LAST2[23]. Qun Chen et al. reported that NEAT1 bind to EZH2 to suppress the Wnt signaling pathway and thereby regulated Glioblastoma progression[49]. Considering that our in vitro cellular model of cardiac fibrosis was stimulated using TGF-β1, and Smad7 is a very classical key antagonist of the reported TGF-β1/Smad2/3 signaling pathway [50, 51], which is normally considered to inhibit Smad2, Smad3, and Smad4 [52], thereby suppressing the progression of many fibrotic diseases, has also been reported to be inhibited by binding to EZH2 [53]. Therefore, we chose Smad7 as a downstream molecule for this study. Accordingly, our experiments showed that GSK126 (an inhibitor of EZH2) attenuated the TGF-β1-induced downregulation of Smad7 expression and the upregulation of p-Smad2/3. Further, the ChIP-qPCR experiment demonstrated that Smad7 did bind to EZH2 and H3K27me3 and that the extent of binding was regulated by the expression of Neat1. Furthermore, rescue experiments demonstrated that GSK126 similarly reversed the downregulation of Smad7 expression caused by overexpression of Neat1. Besides, we found that overexpression of Smad7 successfully rescued the progression of cardiac fibrosis caused by overexpression of Neat1. About how NEAT1 regulate the function of EZH2 thus limiting its binding to the smad7 promoter, we really didn't go further in the molecular space structure to study the regulatory relationship between them since NEAT1 does not affect the expression of EZH2. It is widely studied that as an epigenetic modulating protein, EZH2 function can be regulated by several lncRNAs via direct interaction. While the interaction itself usually showed no influence on EZH2 expression and degradation [33, 54], only reported to guide EZH2 to / away from respective promoters of specific genes which was consistent with the results of our original articles. As for how NEAT1 regulate the function of EZH2 thus limiting its binding to the smad7 promoter, we offer the following hypothesizes: According to the molecular characteristics of lncRNA [55, 56], it is likely that a part of single-strand NEAT1 is complimentary to the sequence adjacent to SMAD7 promoter. Thus, NEAT1 could led free EZH2 in nucleus or from other positions on the chromosome to the SMAD7 promoter. Therefore, the present findings imply that EZH2 inhibits the anti-fibrotic function of Smad7 by binding to its promoter region and suppressing its expression, and the extent of this binding is regulated by Neat1. This means that Neat1 and EZH2 might exert their pro-fibrotic effects through suppression of Smad7 expression.

**Fig. 8:**
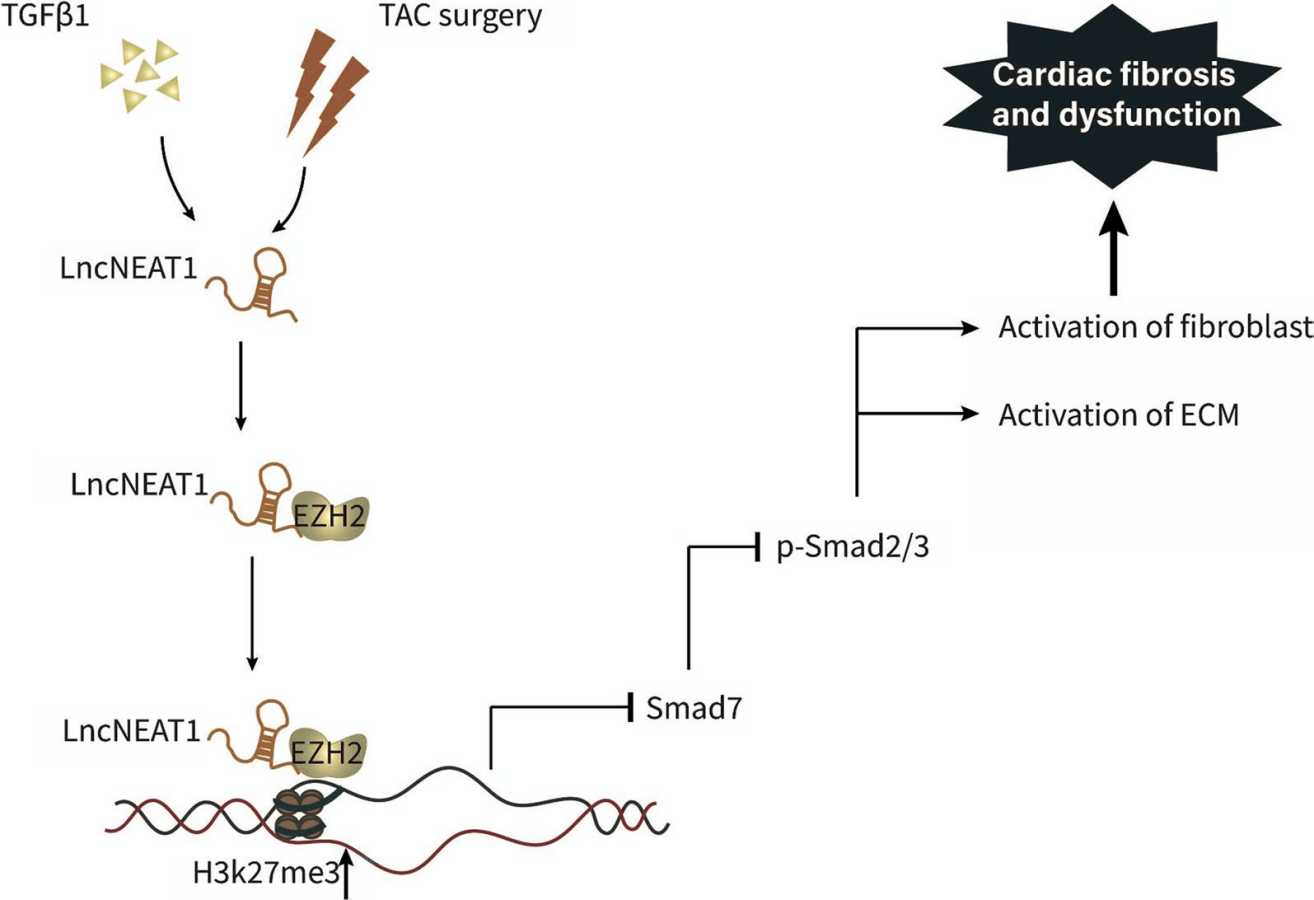
Schematic model of Neat1 regulation of cardiac fibrosis. Neat1 recruits EZH2 to bind to the promoter region of Smad7, thereby inhibiting the expression of Smad7. This leads to the activation of p-Smad2/3, causing the activation of fibroblasts and ECM and ultimately leading to cardiac fibrosis

In conclusion, the present study reveals for the first time the function and mechanism of Neat1 in cardiac fibrosis. NEAT1/Neat1 expression was found to be considerably high in patients with heart failure and mouse models of heart failure. Accordingly, silencing Neat1 significantly alleviated the progression of cardiac fibrosis and dysfunction, while overexpression of Neat1 had the opposite effect. With regard to the underlying molecular mechanism, we found that Neat1 exerted its pro-fibrotic effects by recruiting EZH2 to induce H3K27me3 methylation at the Smad7 promoter, leading to the inhibition of Smad7 and the promotion of cardiac fibrosis (Fig. 8). However, our study has some limitations that need to be resolved in the future. First, in addition to EZH2, other proteins that bind to Neat1 to promote cardiac fibrosis also need to identified. Further, apart from Smad7, other fibrosis-related target genes that might be inhibited by binding to EZH2 also need to be identified. Finally, although we conducted in vivo loss-of-function experiments with AAV9-shRNA in the model mice, it may ultimately be necessary to construct either global knockdown mice or cardiac conditional knockout mice to obtain more convincing evidence in future follow-up studies.

## Supplementary Information

**Additional file 1: Figure S1** A The transfection efficiency of NEAT1 overexpression adenovireus was tested by GFP detection.

**Additional file 2: Figure S2** Alleviation of Neat1-induced cardiac fibrosis, migration, and proliferation in cultured CFs by overexpression of Smad7 **A** CFs were analyzed by immunofluorescence analysis of the expression of α-SMA (red) and nuclei (DAPI: blue) (n = 5 in each group; scale bar = 500 µm). **B** Representative images of Transwell migration assay and quantification of migrated CFs in the indicated groups (n = 5 in each group; scale bar = 100 µm). **C.** Quantification by the CCK-8 assay (n = 5 in each group). **D-F** mRNA levels of Collagen1, Collagen3, and CTGF by qRT-PCR (n = 5 in each group). Data are presented as mean ± SEM. *p < 0.05, **p < 0.01, ***p < 0.001, NS = no significant difference between the indicated groups.

**Additional file 3: Figure S3** Prediction of the potential binding between NEAT1 and EZH2 and the RNA pull-down of EZH2 and NEAT1. **A** Interaction Map of EZH2 and NEAT1, showing a positive result. **B** Interaction probabilities generated by RPISeq range from 0 to 1. In performance evaluation experiments, predictions with probabilities > 0.5 were considered “positive”, indicating that the corresponding RNA (NEAT1) and protein (EZH2) are likely to interact. **C** RNA pull-down results of NEAT1 and EZH2.

## Data Availability

The datasets used and/or analyzed during the current study are available from the corresponding author on reasonable request.
