# Supplementary figures and images for: Long noncoding RNA NEAT1 promotes cardiac fibrosis in heart failure through increased recruitment of EZH2 to the Smad7 promoter region
Source: J Transl Med. 2022 Jan 3;20:7. doi: 10.1186/s12967-021-03211-8 (PMC8722118; doi:10.1186/s12967-021-03211-8)

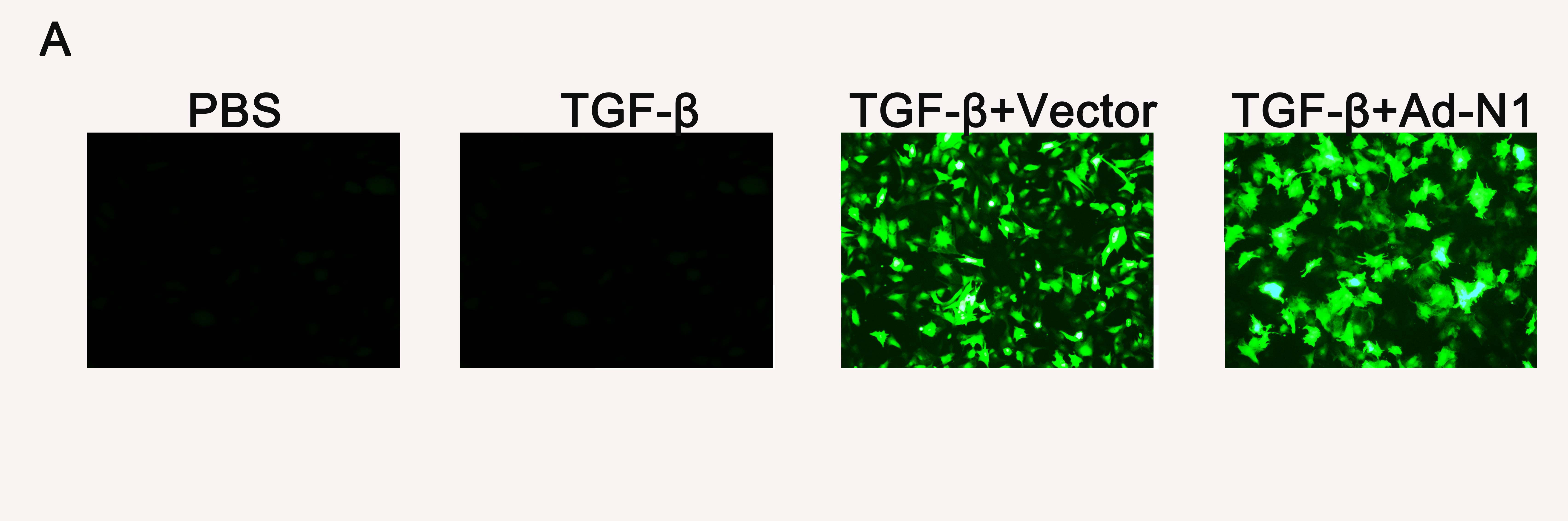

Supplement: Supplementary file 1 — Additional file 1: Figure S1 A The transfection efficiency of NEAT1 overexpression adenovireus was tested by GFP detection. [file 12967_2021_3211_MOESM1_ESM.jpg]

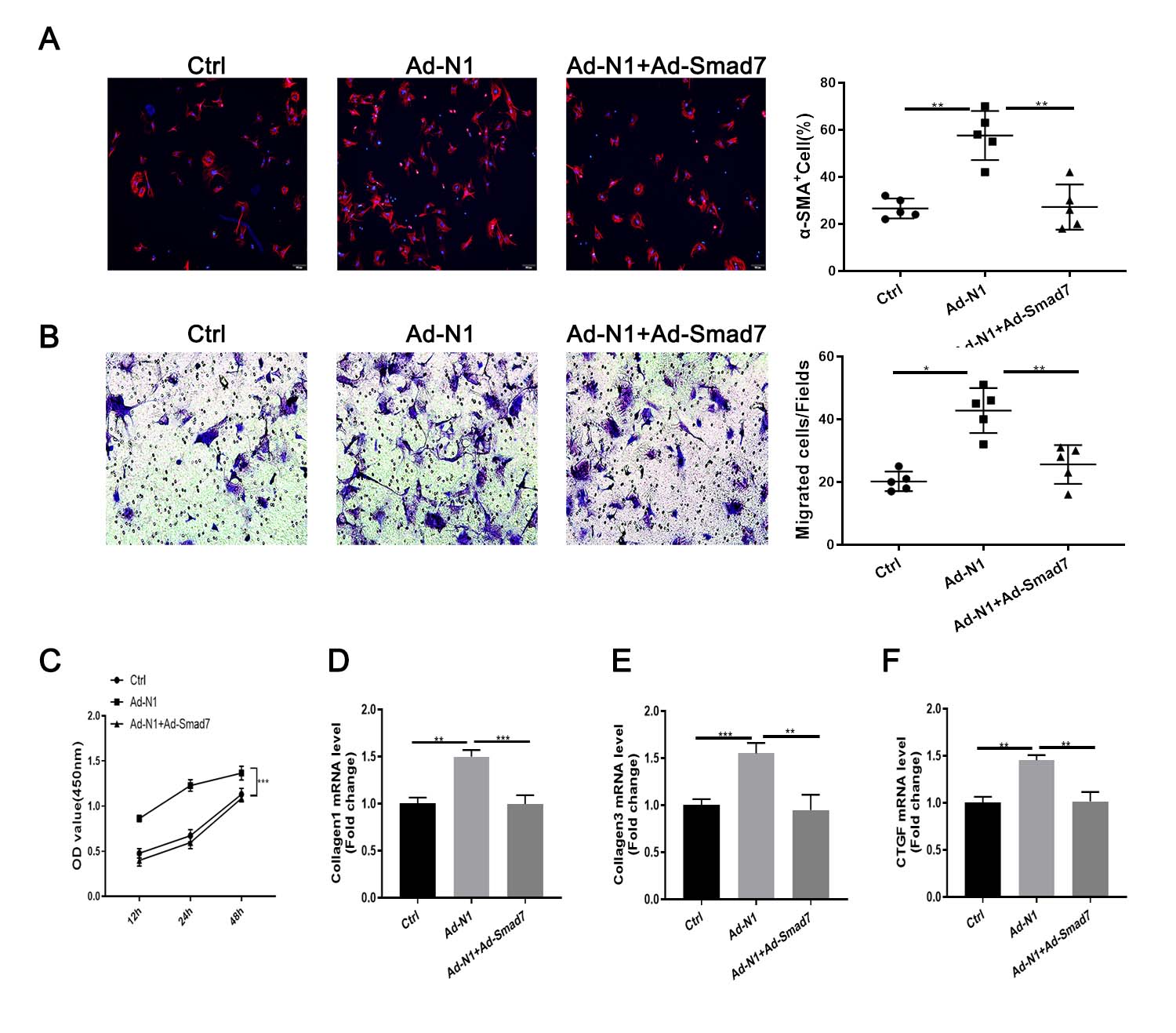

Supplement: Supplementary file 2 — Additional file 2: Figure S2 Alleviation of Neat1-induced cardiac fibrosis, migration, and proliferation in cultured CFs by overexpression of Smad7 A CFs were analyzed by immunofluorescence analysis of the expression of α-SMA (red) and nuclei (DAPI: blue) (n = 5 in each group; scale bar = 500 µm). B Representative images of Transwell migration assay and quantification of migrated CFs in the indicated groups (n = 5 in each group; scale bar = 100 µm). C. Quantification by the CCK-8 assay (n = 5 in each group). D-F mRNA levels of Collagen1, Collagen3, and CTGF by qRT-PCR (n = 5 in each group). Data are presented as mean ± SEM. *p < 0.05, **p < 0.01, ***p < 0.001, NS = no significant difference between the indicated groups. [file 12967_2021_3211_MOESM2_ESM.jpg]

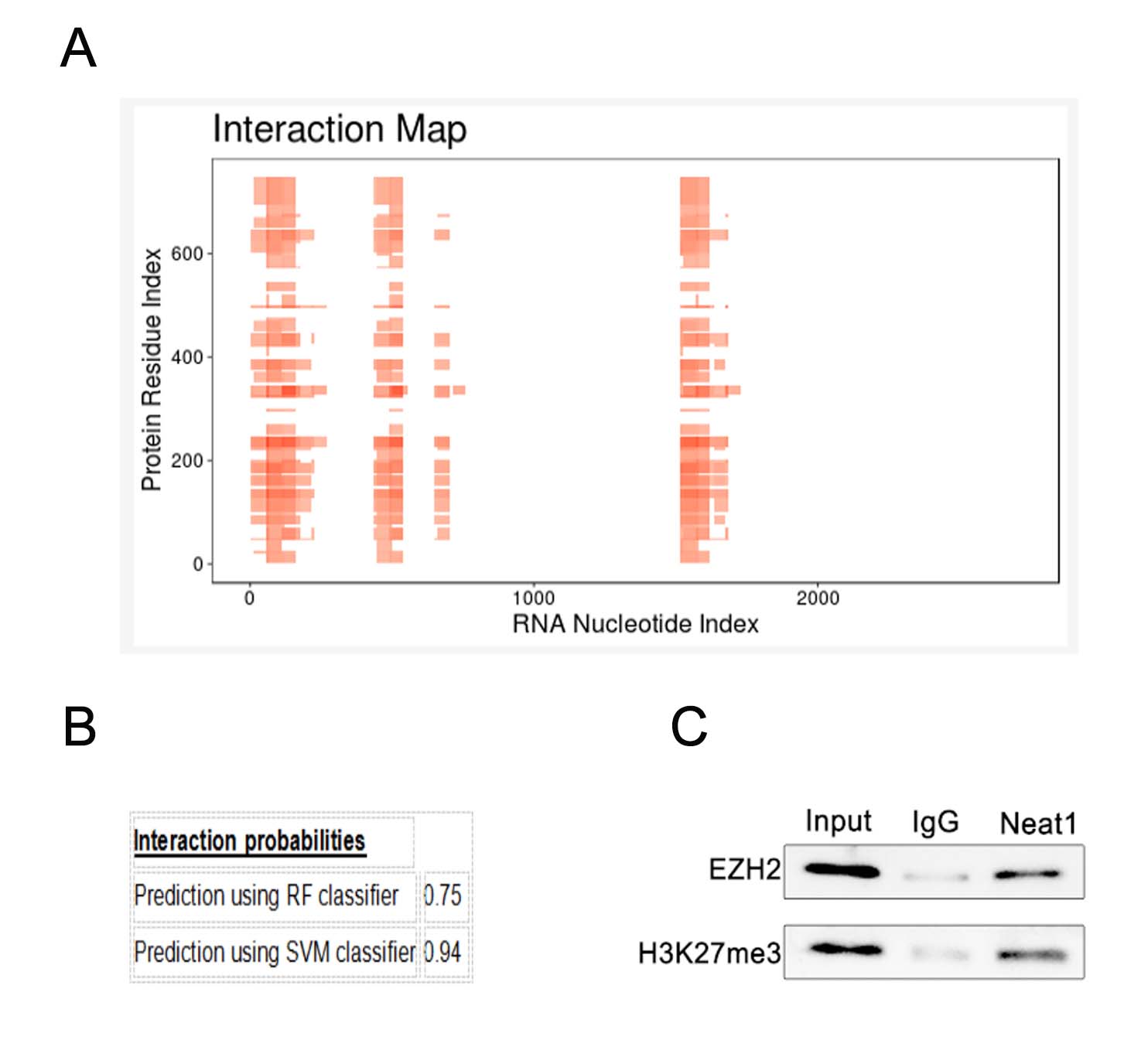

Supplement: Supplementary file 3 — Additional file 3: Figure S3 Prediction of the potential binding between NEAT1 and EZH2 and the RNA pull-down of EZH2 and NEAT1. A Interaction Map of EZH2 and NEAT1, showing a positive result. B Interaction probabilities generated by RPISeq range from 0 to 1. In performance evaluation experiments, predictions with probabilities > 0.5 were considered “positive”, indicating that the corresponding RNA (NEAT1) and protein (EZH2) are likely to interact. C RNA pull-down results of NEAT1 and EZH2. [file 12967_2021_3211_MOESM3_ESM.jpg]
